# Italian Cross-Cultural Adaptation and Validation of the 7-Item Eustachian Tube Dysfunction Questionnaire (ETDQ-7)

**DOI:** 10.1055/a-2873-3749

**Published:** 2026-08-14

**Authors:** Alessandro Marcocci, Antje-Maren Heyer, Maria Emanuela Schiraldi, Roberto Magnato, Charbel Khoury, Barbara Kofler

**Affiliations:** 1 Department of Otorhinolaryngology and Maxillofacial Surgery, Franz Tappeiner Hospital, Meran/Merano, South Tyrol, Italy; 2 Department of Otorhinolaryngology-Head and Neck Surgery, Bozen/Bolzano Hospital, South Tyrol, Italy

**Keywords:** eustachian tube, acoustic impedance tests, ear, middle, surveys and questionnaires

## Abstract

**Introduction:**

Chronic dysfunction of the Eustachian tube is a common complaint, affecting approximately 1–5% of adults and significantly reducing quality of life. This condition may give rise to various symptoms and disorders of the middle ear. To date, the diagnostic tools for this dysfunction remain inadequate. In 2012, McCoul et al. published a questionnaire for the evaluation of Eustachian tube dysfunction, known as the ETDQ-7, and demonstrated its validity and reliability.

**Objectives:**

To prove an in Italian translated version of the ETDQ-7.

**Methods:**

The ETDQ-7 was translated into Italian and administered to two groups: 68 patients with chronic Eustachian tube dysfunction (ETD) and 50 healthy controls. Statistical analyses included test-retest reliability, item–total score correlation, receiver operating characteristic (ROC) analysis, and assessment of internal consistency.

**Results:**

Analysis of the Italian version of the ETDQ-7 confirmed the findings of McCoul et al. (2012). The Cronbach's alpha coefficient was 0.838 for the entire questionnaire. The Spearman's rho coefficient for test–retest was 0.804, indicating good temporal stability in patients' responses. Both total and item scores were significantly higher in the patient group than in the control group.

**Conclusion:**

The Italian version of the ETDQ-7 demonstrates high validity and reliability. Its use is recommended for diagnosing and monitoring patients with chronic Eustachian tube dysfunction.

## Introduction

The Eustachian tube (ET) plays a fundamental role in the ventilation of the middle ear (ME). Alongside patent nasal airways, the ET is crucial for regulating middle ear pressure. 1 Its primary functions include ventilating the ME, protecting against sound pressure and pathogens ascending from the nasopharynx, and draining secretions from the ME into the nasopharynx. Furthermore, the ET equalises rapid pressure changes, thereby protecting both the ME and the tympanic membrane from injury. 2 3

Eustachian tube dysfunction (ETD) can lead to various symptoms, including aural fullness and pressure, tinnitus, and failure of the Valsalva manoeuvre. Chronic ETD is also associated with reduced quality of life.

Several treatment modalities for ETD exist, ranging from conservative approaches, such as the use of decongestant nasal sprays, to surgical interventions, including ET balloon dilatation. 4 5 Accurate diagnosis is essential to ensure appropriate treatment and to distinguish ETD from other ME pathologies.

Numerous diagnostic methods have been proposed to detect ETD; however, their clinical application remains limited. A universally accepted diagnostic test is lacking, and the absence of a gold standard hampers the identification of patients most likely to benefit from treatment. Additionally, assessing baseline symptoms and evaluating treatment outcomes is challenging without a standardised assessment tool. 6

In 2012, McCoul et al. introduced a questionnaire for evaluating ETD-related symptoms: the Eustachian Tube Dysfunction Questionnaire-7 (ETDQ-7). 7 The ETDQ-7 comprises seven questions targeting key symptoms of ETD and is modelled on existing instruments such as the OM-6 8 and SNOT-20. 9 It is a brief, simple, and validated tool that supports the diagnosis of ETD. The questionnaire enables clinicians to characterise ETD symptoms accurately and to distinguish effectively between pathological and normal ET function in adults. To date, the ETDQ-7 has been successfully translated and validated in several languages, including German, Danish, Turkish, Brazilian Portuguese, Greek, and Spanish. 10 11 12 13 14 15 The present study aims to translate and validate the ETDQ-7 in Italian.

## Methods

In this prospective case–control study, patients with eustachian tube dysfunction (ETD) presenting to the Department of Otorhinolaryngology and Maxillofacial Surgery, Franz Tappeiner Hospital Meran/o, were invited to participate. The recruitment period spanned from December 2019 to March 2020. The study endpoint was early March 2020, approximately 5 weeks after the first patient tested positive for COVID-19 and shortly before the first lockdown in Italy. The control group comprised healthy volunteers recruited from individuals undergoing routine examination at our department who exhibited no symptoms of ETD.

Written informed consent was obtained from all participants following a detailed explanation of the study procedures. Prior to any patient enrolment, the study received ethical approval from the Ethics Committee of the Free University of Bozen/Bolzano (Reference number: 89-2019; approval date: 12.12.2019). The study was conducted in full compliance with the principles of the Declaration of Helsinki.

### Patient Selection

A total of 68 patients with chronic ETD and 50 healthy controls were included. The inclusion criterion for both groups was a minimum age of 18 years.

All participants underwent a complete otorhinolaryngological examination, including fibreoptic nasopharyngolaryngoscopy (Storz, Tuttlingen, Germany) and standard tympanometry (Interacoustics, model AZ, Middelfart, Denmark).

In the chronic ETD group, inclusion criteria comprised clinical symptoms such as aural fullness or ear pressure, tinnitus, hearing loss or muffled hearing, and inability to equalise ear pressure, all persisting for more than three months. Additionally, patients were required to exhibit signs of tympanic retraction on otoscopy and/or middle ear effusion with a type B or C tympanogram.

For the control group, only patients without symptoms of ETD and with normal findings on otorhinolaryngological and fibreoptic examination were included. All control participants had a type A tympanogram.

Exclusion criteria included age under 18 years, ETD symptoms of less than three months' duration, and any tympanic membrane lesions (such as granulomas, polyps, or tympanosclerosis) that might interfere with evaluation. Further exclusion criteria included head and neck surgery within the previous three months, prior radiotherapy to the head or neck, a history of head and neck cancer, acute sinonasal infection, adenoid hypertrophy, craniofacial syndromes (e.g., Down syndrome), cleft palate, ciliary dyskinesia, and systemic immunodeficiencies.

### Cross-Cultural Adaptation of the ETDQ-7 Questionnaire into Italian

The cross-cultural adaptation of the ETDQ-7 questionnaire into Italian followed the five-step process recommended by Guillemin et al., 16 which includes: translation, back-translation, review by a translation and back-translation committee, pre-test validation, and score weighting to reflect cultural context.

Initially, the questionnaire was independently translated into Italian by two authors. The concordance of the translations was then reviewed by a professional Italian linguist. A bilingual professor subsequently back-translated the final version into English. No major discrepancies were identified between the original and the back-translated versions, and the translation committee approved the final Italian version.

The Italian ETDQ-7 questionnaire was then administered to five bilingual patients as a pilot test. Feedback and reported difficulties from this group were analyzed and used to refine the final version. All study participants subsequently received the revised Italian version of the ETDQ-7. For the reliability analysis, 18 patients completed the questionnaire again three weeks after the initial administration without having received any treatment in the interim (see Supplementary File for Italian ETDQ-7).

### Procedure and Statistical Analysis

All data were entered into a custom-built FileMaker Pro 16 database. Information was submitted via a WebDirect-based interface (FileMaker Inc., Santa Clara, CA, USA) to facilitate team-wide collaboration.

Descriptive statistics, including mean, median, minimum, and maximum values, standard deviation, and both absolute and relative frequencies (percentages), were used to summarise the data. A two-dimensional scatter plot was also generated. Test-retest reliability of the ETDQ-7 was evaluated using Spearman's correlation coefficient. Internal consistency was assessed using Cronbach's alpha coefficient, and discriminatory validity between patient and control groups was determined via Receiver Operating Characteristic (ROC) curve analysis.

A significance level of 5% was applied for all statistical tests. Data were exported from FileMaker Pro 16 and analyzed using SPSS Statistics, version 24.0 (IBM Corp., Armonk, NY, USA).

## Results

### Patients

A total of 118 participants were enrolled in this study: 68 patients diagnosed with ETD and 50 healthy controls. The mean age (standard deviation) was 53 (±16.75) years in the ETD group and 49 (±13.96) years in the control group. The ETD group included 19 men (27.9%) and 49 women (72.1%), while the control group consisted of 22 men (44%) and 28 women (56%).

### Internal Consistency Reliability

Internal consistency of the ETDQ-7 was high, with a Cronbach's alpha (α) of 0.838 for the entire instrument (95% confidence interval: 0.799–0.837). Evaluation of internal consistency with individual items removed did not result in any substantial improvement ( Table 1 ).

**Table 1:** Reliability of the ETDQ-7: Overall Internal Consistency and Internal Consistency with Each Item Removed

Item	Cronbach's Alpha if Item Removed
1. Pressure	0.799
2. Pain	0.832
3. Feeling clogged	0.802
4. Cold/sinusitis problems	0.837
5. Crackling or popping	0.802
6. Ringing	0.819
7. Feeling muffled	0.814
Overall	**0.838**

### Test-Retest Reliability

Eighteen patients with ETD completed the ETDQ-7 a second time, three weeks after the initial administration, without having received any treatment. The test–retest reliability was good, with a Spearman's rho coefficient of 0.804, indicating a strong correlation between responses over time.

### Discriminant Validity

The overall ETDQ-7 score among the 68 ETD patients was significantly higher than that of the 50 control participants (t = 25.64, p < 0.001). The mean (standard deviation) total score was 3.55 (1.07) for the ETD group and 1.30 (0.29) for the control group. Each of the seven items on the ETDQ-7 demonstrated significantly higher scores in the ETD group, with a correlation of 97.9% and p < 0.001 ( Table 2 ).

**Table 2:** Eustachian Tube Dysfunction Questionnaire Scores by Item

Item	ETD Group Mean (SD)	Control Group Mean (SD)
1. Pressure	3.69 (1.84)	1.20 (0.49)
2. Pain	2.77 (1.93)	1.10 (0.36)
3. Feeling clogged	4.45 (1.83)	1.43 (0.61)
4. Cold/sinusitis problems	3.41 (2.07)	1.55 (1.13)
5. Crackling or popping	3.35 (2.10)	1.34 (0.62)
6. Ringing	3.04 (2.14)	1.26 (0.77)
7. Feeling muffled	4.14 (2.03)	1.41 (0.73)
**Overall**	**3.55 (1.07)**	**1.30 (0.29)**

ETD, Eustachian tube dysfunction; SD, standard deviation.

Note: All between-group comparisons were performed using the paired t-test (p < 0.001 for all items).

**Fig. 1:**
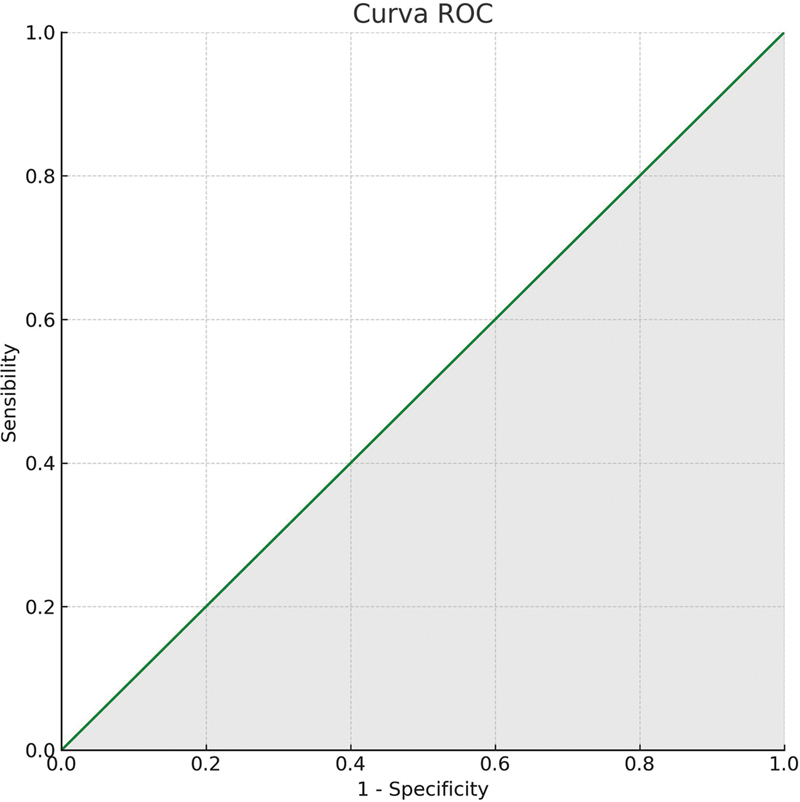
ROC curve for the ETDQ-7 in detecting Eustachian tube dysfunction. Area under the curve (AUC) = 1.0 (p < 0.0001).

Receiver Operating Characteristic (ROC) analysis confirmed excellent discriminant validity of the ETDQ-7 ( Fig. 1 ). Using a cut-off total score of ≥ 14.5 to classify a patient as having ETD yielded a specificity of 100% and a sensitivity of 99.6%, with a positive predictive value of 97.14% and a negative predictive value of 100% ( Fig. 1 ).

## Discussion

Eustachian tube dysfunction (ETD) has an estimated prevalence of 1–5% in the adult population. Despite its frequency, there remains a lack of objective methods to detect and quantify the severity of chronic ETD. Numerous diagnostic tools have been proposed to assess ET function, including audiometry, tympanometry, otoscopy, visual classification of endoscopic findings, and tubomanometry. However, the clinical utility of these tests remains limited, and clinical history and physical examination continue to play a fundamental role in diagnosing ETD.

In recent years, patient-reported outcome measures have gained importance for assessing the impact of disease and treatment on health-related quality of life. In this context, McCoul et al. developed a symptom-based score as a reliable, disease-specific tool to quantify obstructive ETD symptoms. 7 The ETDQ-7 offers several advantages over a traditional clinical history: it provides a more precise estimate of disease burden and yields formal, validated documentation of patient-reported symptoms. Additionally, it is more suitable for therapy monitoring than open-ended anamnesis. Based on these benefits, we translated the ETDQ-7 into Italian and assessed its validity and reliability.

The internal consistency of the ETDQ-7 in our sample was high, with a Cronbach's alpha of 0.838, confirming that the items measured a consistent underlying construct. Receiver operating characteristic (ROC) analysis yielded an area under the curve (AUC) of 1.0 (95% CI: 0.999-1.0), confirming excellent discriminative power between patients with ETD and healthy controls. Test–retest reliability over time was good, with a Spearman's rho coefficient of 0.804, indicating high temporal stability.

Our findings are consistent with those reported by McCoul et al., Schröder et al., Van Roeyen et al., Özgür et al., Pires Gallardo et al., Ikeda et al., Herrera et al. 7 10 12 13 15 17 18 As noted by other authors, 12 we observed that some patients experienced difficulty responding to item 4, particularly when simultaneously affected by nasal or sinus conditions. In such cases, it may be helpful to provide a brief explanation or footnote, as proposed by Özgür et al. for the Turkish version. 12

Ikeda et al. investigated the use of the ETDQ-7 in patients with patulous ET. 17 As the questionnaire does not include items related to aural fullness and autophony, 17 18 it is not suitable for distinguishing between obstructive or patulous ETD nor for measuring the severity of patulous ET symptoms. It is, therefore, essential to identify patients with patulous ET prior to administering the ETDQ-7 to avoid misclassification and the potential inappropriate use of balloon Eustachian tuboplasty based on misleadingly high ETDQ-7 scores.

In our study group, the majority of patients diagnosed with ETD were female (72.1% versus 27.9%). Oehlandt et al.19 investigated 432 patients (41% male, 59% female) and did not find a significant association between ETD and patient characteristics. The authors found that sex was not associated with tubomanometry results. However, female sex was associated with higher ETDQ-7 scores (p = 0.030). Additionally, the odds of female patients being unable to perform the Valsalva manoeuvre were 1.97 times higher than those of male patients (95% CI: 1.29-3.02, p = 0.002). 19

Alshamani et al. 20 also investigated in 380 patients the role of sex in ETD patients diagnosed via the ETDQ-7. The authors found sex to be significantly associated with the prevalence of ETD (p = 0.004); ETD was detected more frequently among female patients.

In the present study, sex-specific differences were not explicitly investigated. Nevertheless, the observed gender distribution and previously reported associations in the literature suggest that future studies specifically designed to explore potential gender-related differences in ETD presentation and questionnaire performance may provide valuable additional insights.

The results of this study confirm the usefulness of the ETDQ-7 as a diagnostic aid for obstructive ETD. Nonetheless, it should be used in conjunction with clinical findings and audiotympanometry to establish a comprehensive diagnosis, encompassing dilatory, baro-challenge-induced, and patulous ETD, as recommended in the consensus guidelines by Schilder et al. 21

## Conclusion

This study demonstrates that the ETDQ-7 is a reliable and precise tool for assessing symptoms in patients with obstructive ETD and is, therefore, a valuable instrument in clinical practice. However, it does not differentiate between obstructive and patulous ETD, and its responsiveness to treatment-related changes in symptom severity has yet to be fully evaluated.

Disease-specific questionnaires such as the ETDQ-7 can serve as an important outcome measure for clinical interventions. Key attributes contributing to their validity include responsiveness, sensitivity to clinical change, and criterion validity. A prospective study involving a larger cohort is warranted to further assess the utility of the ETDQ-7 in monitoring treatment outcomes following medical or surgical management of ETD.
